# An *Acinetobacter* trimeric autotransporter adhesin reaped from cells exhibits its nonspecific stickiness via a highly stable 3D structure

**DOI:** 10.1038/srep28020

**Published:** 2016-06-16

**Authors:** Shogo Yoshimoto, Hajime Nakatani, Keita Iwasaki, Katsutoshi Hori

**Affiliations:** 1 Department of Biotechnology, Graduate School of Engineering, Nagoya University, Furo-cho, Chikusa-ku, Nagoya, Aichi 464-8603, Japan

## Abstract

Trimeric autotransporter adhesins (TAAs), cell surface proteins of Gram-negative bacteria, mediate bacterial adhesion to host cells and extracellular matrix proteins. However, AtaA, a TAA in the nonpathogenic *Acinetobacter* sp. strain Tol 5, shows nonspecific, high adhesiveness to abiotic material surfaces as well as to biotic surfaces. AtaA is a homotrimer of polypeptides comprising 3,630 amino acids and forms long nanofibers; therefore, it is too large and structurally complex to be produced as a recombinant protein. In this study, we isolated AtaA’s passenger domain (AtaA PSD), which is translocated to the cell surface through the C-terminal transmembrane domain and exhibits biological functions, using a new method. We introduced a protease recognition site and reaped AtaA nanofibers 225 nm in length from the cell surface through proteolytic cleavage with a specific protease. Biochemical and biophysical analyses of the purified native AtaA PSD revealed that it has a stable structure under alkaline and acidic conditions. Temperatures above 80 °C, which disrupted AtaA’s higher-order structure but maintained the full-length AtaA polypeptide, inactivated AtaA’s nonspecific adhesiveness, suggesting that the stickiness of AtaA requires its 3D structure. This finding refutes the widespread but vague speculation that large unfolded polypeptides readily stick to various surfaces.

In autotransporters, extracellular proteins in diverse Gram-negative bacteria, the transmembrane domain (TM) hosts the autotransport function, also called type V secretion^1^. During this process, the passenger domain (PSD) is exported to the bacterial cell surface through a pore formed by the TM, with the assistance of periplasmic chaperones and the β-barrel assembly machinery^2^. Although classical autotransporters comprise one polypeptide with a signal peptide, a PSD at the N-terminus of the mature protein, and a C-terminal TM, trimeric autotransporter adhesins (TAAs) form homotrimeric structures with a common N-terminus–head–stalk–TM–C-terminus architecture after eliminating a signal peptide^3^,^4^,^5^. The TM, which is formed by a 12-stranded β-barrel (four strands from each polypeptide × three polypeptides) at the outer membrane (OM), anchors the head–stalk domain, the PSD of TAAs, to the OM, after secreting it through the β-barrel pore. The PSDs are assembled from a set of analogous, structurally conserved domains, and the evolutionary rearrangement of these domains has resulted in considerable diversity in the configuration and length of PSDs^4^. Most TAAs have been reported to be involved in the initial attachment of bacterial cells to host cells and/or extracellular matrix proteins, as well as in autoagglutination, colonization, biofilm formation, and serum resistance^6^,^7^,^8^,^9^,^10^,^11^,^12^,^13^,^14^,^15^,^16^,^17^,^18^,^19^. The TAAs in *Burkholderia* species are currently attracting attention for their ability to drive actin-based motility in host cells by mimicking host actin polymerases^20^. The PSDs are responsible for these biological functions.

AtaA, a TAA from the nonpathogenic toluene-degrading bacterium *Acinetobacter* sp. Tol 5^21^, has unique adhesive properties that are not shared by other reported TAAs; AtaA mediates strikingly tenacious, nonspecific adhesion of the bacterial cells to abiotic surfaces and has dramatically high autoagglutination ability^22^. Through AtaA’s adhesive properties, large clumps of the bacterial cells are rapidly immobilized onto various material surfaces, ranging from hydrophobic plastics to hydrophilic glass and stainless steel, in a manner independent of cell growth^23^. A novel and highly efficient method for microbial cell immobilization using AtaA has been developed, and its effectiveness has been demonstrated^24^,^25^. AtaA is composed of 3,630 amino acids and is one of the largest TAAs reported to date, with a long PSD consisting of mosaically arranged multiple domain repeats that often appear in the TAA family and are annotated by the daTAA program^26^. These domain repeats include two Ylheads, 12 Trp-rings, eight DALL1s, five FGG motifs, and several neck domains^27^. This long PSD forms the long stalk of the AtaA nanofiber, which extends for more than 200 nm on the Tol 5 cell surface^22^,^28^. To unravel the mechanism of the striking adhesive properties of AtaA, direct characterization of the AtaA molecule using purified protein is indispensable. The effects of other factors, including the surface charge and hydrophobicity of cells and other nanofibers on the cell surface^28^, can be eliminated using purified protein. However, it is virtually impossible to produce the entire multi-domain AtaA membrane protein as a recombinant protein in a correctly folded state. Alternatively, extensive experiments would be required to find a suitable method for disruption of the membrane envelope and to select the optimum surfactant for the purification of full-length, native AtaA from Tol 5 cells.

In contrast with TAAs, which are anchored to the OM, many classical autotransporters release their PSDs into the extracellular space by proteolytic cleavage, using either autoproteolytic activity or a membrane-bound protease^29^,^30^,^31^,^32^,^33^,^34^,^35^. Furthermore, heterologous passenger proteins fused to the TMs of classical autotransporters have been released to the external environment by OmpT protease^29^,^36^. An OmpT protease recognition site was also inserted at the fusion site of a heterologous epitope with the TM to release the heterologous passenger polypeptide by proteolytic cleavage^37^.

Here, we show the first example of applying proteolytic cleavage to a TAA for the isolation of its PSD. A specific protease site was artificially introduced to obtain the full-length extracellular portion of AtaA, which forms nanofibers. Long AtaA PSD nanofibers were successfully reaped directly from the cell surface and used to characterize the AtaA molecule.

## Results

### Molecular engineering to obtain the AtaA passenger domain from the cell surface

**Figure 1:**
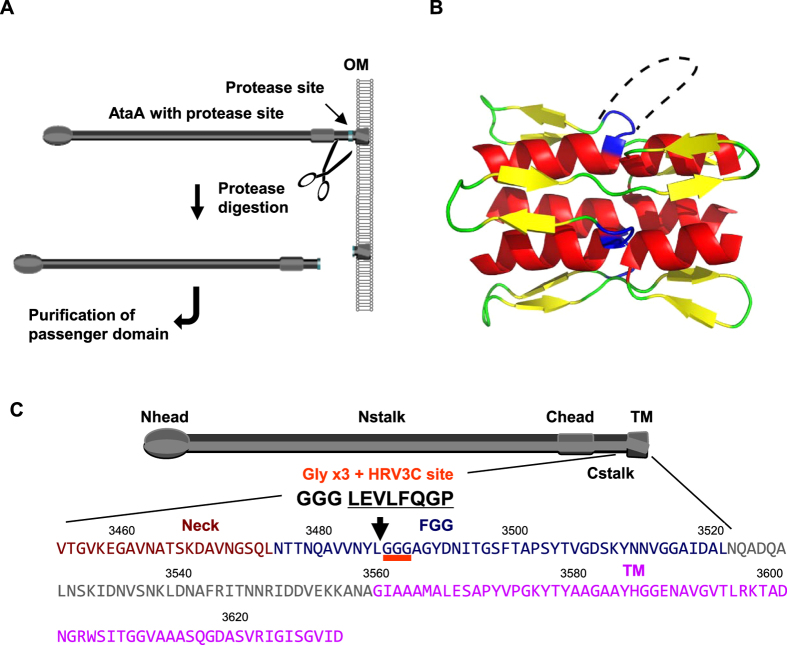
Enzymatic reaping of AtaA passenger domain nanofibers from the cell surface by proteolytic cleavage. (**A**) Scheme for the isolation of AtaA PSD from the cell surface by enzymatic reaping. A specific protease recognition site was genetically introduced at the base of AtaA PSD. The resulting genetically modified AtaA was produced in Tol 5 4140 Δ*ataA* mutant cells and isolated by digestion with the specific protease. (**B**) Molecular design for insertion of the cleavage site by HRV 3C protease into the FGG motif of the AtaA nanofiber. The triglycine loops are shown as blue wires and one of the deduced loops formed by the insertion of the protease recognition sequence is represented by a dotted line. (**C**) The position for the insertion of the triglycine linker plus HRV 3C protease recognition sequence in the AtaA nanofiber and its surrounding amino acid sequence. HRV 3C protease cleaves the recognition sequence between glutamine and glycine. The TM, FGG domain, Neck domain, and the original triglycine residues in loops are shown in magenta, dark blue, and brown, and with red underlining, respectively. The HRV 3C protease recognition sequence (underlined) and triglycine linker were inserted at the position indicated by an arrow.

**Figure 2:**
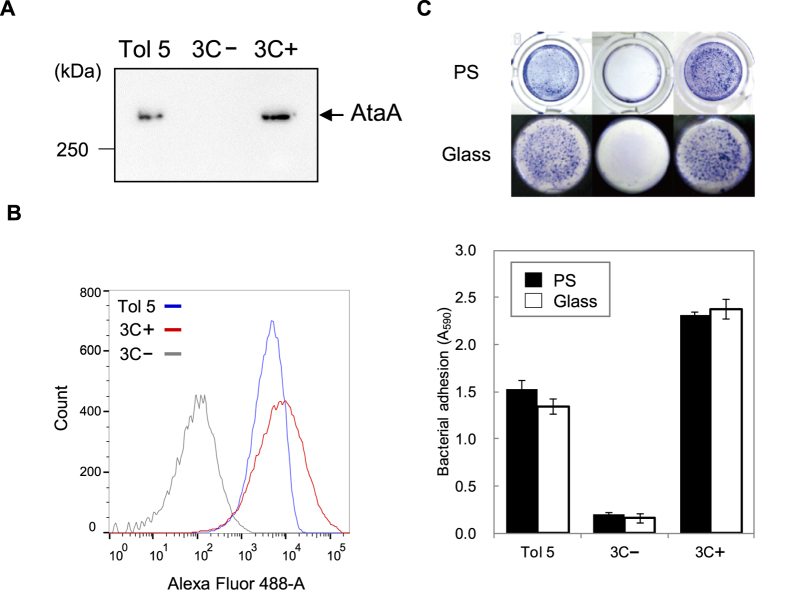
Production and cell surface display of 3CAtaA protein by *Acinetobacter* sp. Tol 5 4140 transformed with p3CataA and the adhesiveness of the transformant. (**A**) Immunoblotting of whole-cell lysates with anti-AtaA_699–1014_ antiserum. (**B**) Flow cytometric analysis using anti-AtaA_699–1014_ antiserum for labeling and Alexa Fluor 488-conjugated secondary antibody. (**C**) Adherence assay using polystyrene (PS) and glass plates. Cells adhering to the surfaces were visualized with crystal violet staining. The graph shows quantified data of cells adhering to PS (filled bars) and glass (blank bars) surfaces by measuring the A_590_ of the extracted stain. Data are expressed as the mean ± SEM of three independent assays. In (**A**,**B**,**C)** Tol 5, Tol 5 wild-type; 3C−, Tol 5 4140 (p3CataA) without induction of *3CataA* gene expression; 3C+, Tol 5 4140 (p3CataA) with induction of *3CataA* gene expression.

We designed an isolation method that enables reaping of the PSD nanofiber directly from the cell surface by cleaving the AtaA protein at the base of the PSD using a protease with high specificity (Fig. 1). According to the annotation provided by the daTAA program, AtaA has five FGG motifs, which are numbered from the N-terminus of AtaA (FGG_1 to FGG_5); FGG_5 is situated at the N-terminal side adjacent to the TM. A previously determined X-ray crystal structure that included FGG_5 (PDB ID = 3WPA) showed that it has a tri-glycine loop exposed on the molecular surface of the fiber (Fig. 1B)^27^. We selected a position between the leucine residue and the tri-glycine residues in this FGG motif to insert the protease cleavage site so as to form an expanded flexible loop. By digesting at this position, 98.5% of the full-length fiber of the trimeric AtaA PSD, which contains 3428 amino acids in each polypeptide, would be reaped from the cell surface. It is 1016 kDa as the trimer based on the calculated molecular weight and has a theoretical isoelectric point of 4.76. For the site-specific cleavage of the PSD, we selected human rhinovirus 3C (HRV 3C) protease, which recognizes a unique amino acid sequence with high specificity. A nucleotide sequence encoding the protease recognition site and a tri-glycine linker was inserted into the full-length *ataA* gene at the target site (Fig. 1C). The resultant recombinant DNA encoding the modified AtaA (3CAtaA) was used to transform the Δ*ataA* mutant strain Tol 5 4140^38^ for its exclusive expression. Production of 3CAtaA protein and its cell-surface display were analyzed by immunoblotting and flow cytometry, respectively. Immunoblotting analysis of the whole-cell lysates demonstrated the production of 3CAtaA protein in Tol 5 4140 (p3CataA) cells with induction of *3CataA* gene expression by arabinose (3C+) but not in the cells without the induction (3C−) (Fig. 2A). Flow cytometric analysis using cells stained with anti-AtaA antiserum revealed that Tol 5 4140 (p3CataA) cells with induction of *3CataA* gene expression exhibited a shift of the histogram toward the stronger fluorescent side, implying much 3CAtaA protein displayed on the cell surface, compared with even surface display of AtaA protein on Tol 5 wild-type cells. The functionality of 3CAtaA protein on the cell was examined by adherence assays using polystyrene (PS) and glass plates. As shown in Fig. 2C, cells expressing 3CAtaA (3C+) exhibited higher adhesion to both material surfaces than not only cells without expressing *3CAtaA* (3C−) but also Tol 5 wild-type cells expressing the original AtaA, due to higher expression of 3CataA from the plasmid than that of AtaA from the genome. These results verified that 3CAtaA was properly displayed on the cell surface and kept the adhesiveness of the original AtaA in spite of the insertion of the HRV 3C recognition site.

### Reaping AtaA PSD nanofibers from the cell surface by proteolytic cleavage

**Figure 3:**
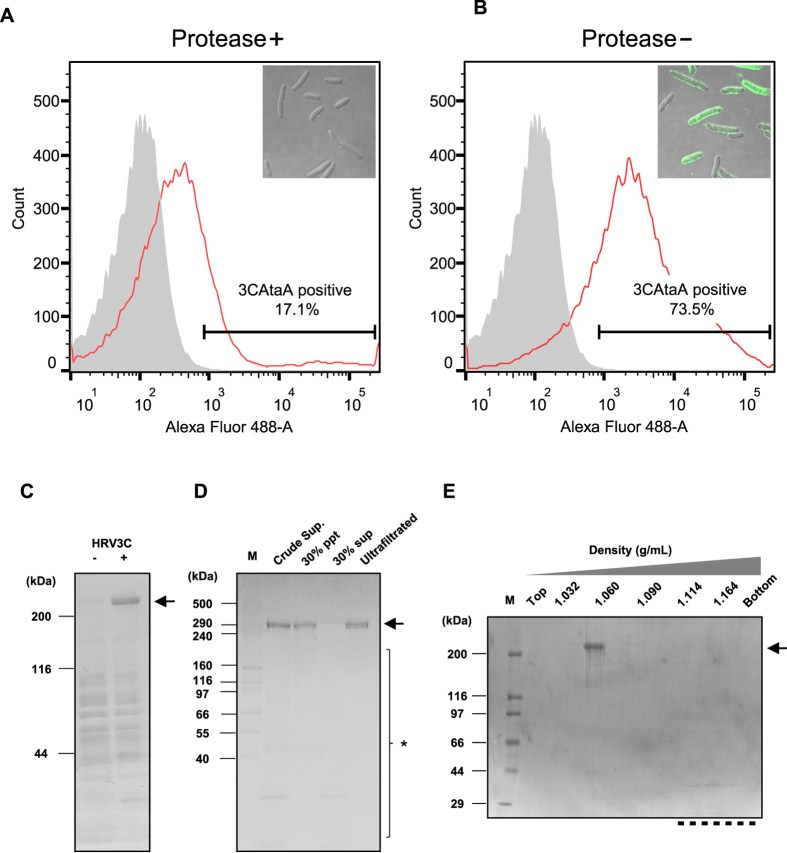
Isolation and purification of AtaA PSD from the cell surface through HRV 3C protease treatment. (**A,B**) Flow cytometric quantification of AtaA PSD nanofibers remaining on Tol 5 4140 transformant cells expressing *3CataA* after treatment with (+) or without (−) HRV 3C protease. Insets, CLSM images of the cells stained with anti-AtaA antiserum and Alexa Fluor 488-conjugated secondary antibody after reaping AtaA PSD nanofibers. (**C**) SDS-PAGE and Coomassie Brilliant Blue (CBB) staining of supernatant samples after treatment with (+) or without (−) HRV 3C protease. (**D**) SDS-PAGE and CBB staining of the supernatant sample after precipitation in the 30% solution of the saturated concentration of ammonium sulfate. M, marker lane; Crude Sup, supernatant (sup) sample; 30% ppt and 30% sup, insoluble precipitate (ppt) and soluble sup fractions, respectively, from ammonium sulfate precipitation in the 30% solution of its saturated concentration; Ultrafiltrated, the concentrated sample from the 30% ppt. fraction using a 100 kDa cut-off ultrafiltration membrane. (**E**) Purification of AtaA PSD from the protein sample precipitated in the 30% solution of the saturated concentration of ammonium sulfate in (**D**) by 5–50% of sucrose density gradient centrifugation. A dotted line represents high-density fractions that contain OM. M, marker lane; Top and Bottom, lowest- and highest-density fractions. Density values of fractions (g/mL) are indicated on the lanes. An arrow in each panel indicates bands corresponding to AtaA PSD.

Tol 5 4140 transformant cells, which express 3CAtaA, were treated with HRV 3C protease to specifically cleave AtaA present on the cell surface. After the proteolytic digestion, the cells were harvested by centrifugation to examine the cleavage efficiency, and the supernatant was collected for the purification of AtaA PSD released in the reaction buffer. The cells treated with or without the protease were stained with anti-AtaA antiserum and observed under a confocal laser scanning microscope (CLSM). As shown in insets of Fig. 3A,B, treatment of protease drastically reduced the fluorescence of the cells, implying effective cleavage of AtaA displayed on the cells. For more quantitative evaluation of the proteolytic cleavage efficiency, we performed flow cytometric analysis using the same samples as used for CLSM. The protease treatment reduced the percentage of the fluorescent labeled cells displaying 3CAtaA from 73.5% (Fig. 3B) to 17.1% (Fig. 3A). Therefore, the cleavage efficiency was calculated to be 77%.

As shown in Fig. 3C, AtaA PSD could be recovered from the supernatant of the sample treated with the protease but could not be recovered from the control sample that did not undergo protease treatment. However, other proteins were present as contaminants in the supernatant, even though it contained mainly AtaA PSD. For further purification, the proteins in the supernatant were precipitated in the 30% solution of the saturated concentration of ammonium sulfate. The majority of the AtaA PSD was recovered in the precipitate with high purity (Fig. 3D). To confirm that the AtaA PSD recovered from the precipitate was dissociated from the OM, a sample was subjected to sucrose density gradient ultracentrifugation, which has been used to purify the OM^39^,^40^,^41^. Upon fractionation on a 5–50% sucrose density gradient, the purified AtaA PSD was found mainly in the fraction with a density of 1.060 g/mL. Little AtaA was detected in the high-density fractions or in the bottom fraction, where OM should have been distributed (Fig. 3E). Thus, AtaA PSD could be isolated *en bloc* from the surface of cells displaying 3CAtaA by proteolytic digestion with HRV 3C protease and could be purified simply by ammonium sulfate fractionation. The AtaA PSD protein in the sample after ammonium sulfate fractionation was concentrated by ultrafiltration with a 100-kDa cut-off filter. We ultimately obtained 5 μg of purified AtaA PSD from 20 mL of culture solution.

### Morphological characteristics of the purified AtaA passenger domain

**Figure 4:**
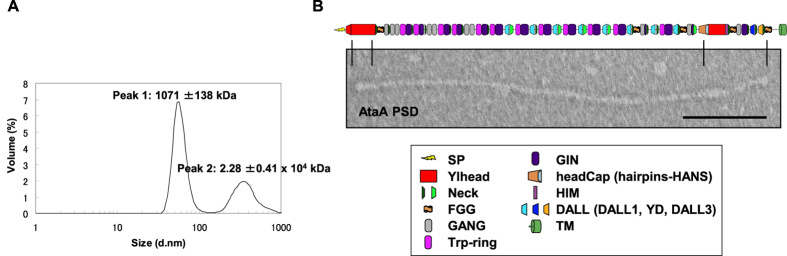
Analysis of the intact fiber structure of AtaA PSD. (**A**) The size distribution of AtaA PSD measured using DLS. (**B**) TEM image of AtaA PSD isolated by our enzymatic reaping method from Tol 5 4140 (p3CataA) cells. The fiber sample was negatively stained before TEM. A schematic of the predicted domain structure of AtaA is depicted above its electron micrograph. Scale bar: 50 nm.

Dynamic light scattering (DLS) analysis was performed to confirm that the purified AtaA PSD maintained its trimeric structure. DLS analysis is often used to determine the size and molecular weight of protein. The molecular weight of the AtaA PSD, modeled as a linear polymer, was estimated to be 1071 ± 138 kDa (Fig. 4A, Peak1). This value almost coincides with the value (1016 kDa) calculated using its constituent amino acids. A peak detected at very high molecular mass might reflect the aggregation of AtaA PSD molecules in the sample (Fig. 4A, Peak2).

We next analyzed the morphological characteristics of AtaA PSD using transmission electron microscopy (TEM). Many nanofibrous structures were observed in the fraction of purified AtaA PSD. AtaA PSD nanofibers were uniform in size, at 225 ± 1.7 nm in length and 4 ± 0.4 nm in thickness (mean ± SEM of 10 fibers, chosen at random), were curved in places, and had two globular structures: one at the fiber tip and the other near the opposite end (see Supplementary Figs S1 and S2). The former globular structure was predicted to correspond to the globular tip of the AtaA nanofiber, that is, N-terminal Ylhead (Nhead). To confirm the topological direction of the nanofibers observed, we constructed another modified AtaA, 3CFGG2AtaA, in which a HRV 3C protease recognition site was inserted at FGG_2 (see Supplementary Fig. S1). After proteolytic cleavage, a shorter version of the AtaA PSD fiber (AtaA PSD short), which did not include the C-terminal Ylhead (Chead), was obtained. TEM revealed that AtaA PSD short fibers were 180 ± 1.8 nm in length (mean ± SEM of six fibers, chosen at random), had only one globular structure at the fiber tip, and lost the other globular structure (see Supplementary Fig. S1). These data suggest that the globular structures at the fiber tip and near the opposite end corresponded to Nhead and Chead, respectively. The TEM image of a single-molecule fiber of AtaA PSD was then aligned to the predicted domain configuration (Fig. 4B).

### Biophysical and biochemical characterization of the AtaA passenger domain

**Figure 5:**
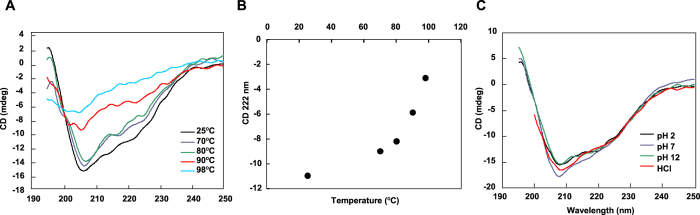
Effect of temperature and pH on secondary structure of AtaA PSD measured by CD spectroscopy. (**A**) CD spectra of AtaA PSD after thermal treatment at various temperatures for 5 min. The measurement was carried out at a room temperature. (**B**) Ellipticity at 222 nm as a function of temperature. (**C**) CD spectra of AtaA PSD in acid or alkaline. The protein samples dissolved in buffer solutions at different pH or in 0.1 M HCl were directly measured by CD spectroscopy.

The effects of temperature and pH on the structure of AtaA PSD were examined by measuring the circular dichroism (CD) spectra. CD is commonly used to estimate the secondary structure of protein. This measurement is widely used to determine the stability of the structure of a protein in various physicochemical conditions. After incubation of AtaA PSD dissolved in deionized water (dH_2_O) at temperatures 25–98 °C for 5 min, CD spectra were measured at a room temperature (Fig. 5A). The CD spectrum of the native AtaA PSD exhibited two negative peaks at 209 and 222 nm that were attributed to α-helices, and one negative peak at 218 nm that was attributed to β-strands; their contents in this intact AtaA PSD were 31% and 10%, respectively. The AtaA PSD treated at 70 and 80 °C showed CD spectra with only small differences from native AtaA PSD at 25 °C. However, the CD spectrum of AtaA PSD changed drastically after incubation at temperatures over 90 °C; The CD signals became weaker, implying the disruption of secondary structures. The ellipticity of the CD peak at 222 nm gradually decreased with increasing temperature below 70 °C, but it drastically decreased above 80 °C, reflecting the disruption of secondary structures within AtaA PSD (Fig. 5B). Next, to examine the effect of pH, the CD spectra of AtaA PSD dissolved in buffers at different pH or in 0.1 M HCl (pH 1.0) were directly measured. The CD spectra of all samples at pHs from 2 to 12, and even in 0.1 M HCl, almost overlapped, implying that the secondary structure of AtaA PSD is mostly unaffected by pH change in this range (Fig. 5C).

**Figure 6:**
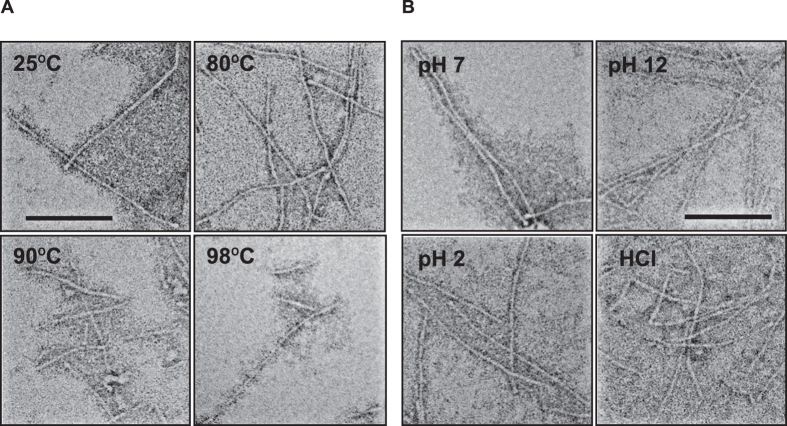
Effect of temperature and pH on the fibrous morphology of AtaA PSD observed by TEM. (**A**) TEM images of AtaA PSD after thermal treatment at various temperatures for 10 min. After the treatment, the samples were subjected to negative staining for TEM. Scale bars: 100 nm. (**B**) TEM images of AtaA PSD treated in different pH solutions. After the treatment, the sample was subjected to negative staining for TEM. Scale bars: 100 nm.

We also examined the morphological changes that occurred in AtaA PSD nanofibers using the same samples used for CD spectroscopy after thermal or pH treatment. TEM revealed no morphological changes resulting from the treatment at 80 °C, relative to the morphology in the control treated at 25 °C. However, the fibrous structure of AtaA PSD shrank, shortened, and was difficult to observe upon thermal treatment at 90 and 98 °C (Fig. 6A), which correspond to temperatures that caused drastic changes in the CD spectrum (Fig. 5A,B). Apparent disruption of the fiber structure was not observed for any pH conditions (Fig. 6B), as expected from the unchanged CD spectra under acidic and alkaline conditions (Fig. 5C). Even in 0.1 M HCl, AtaA PSD maintained a distinctly visible fiber morphology, as well as its secondary structure, although bent fibers were observed.

### Functional analysis of the AtaA passenger domain

**Figure 7:**
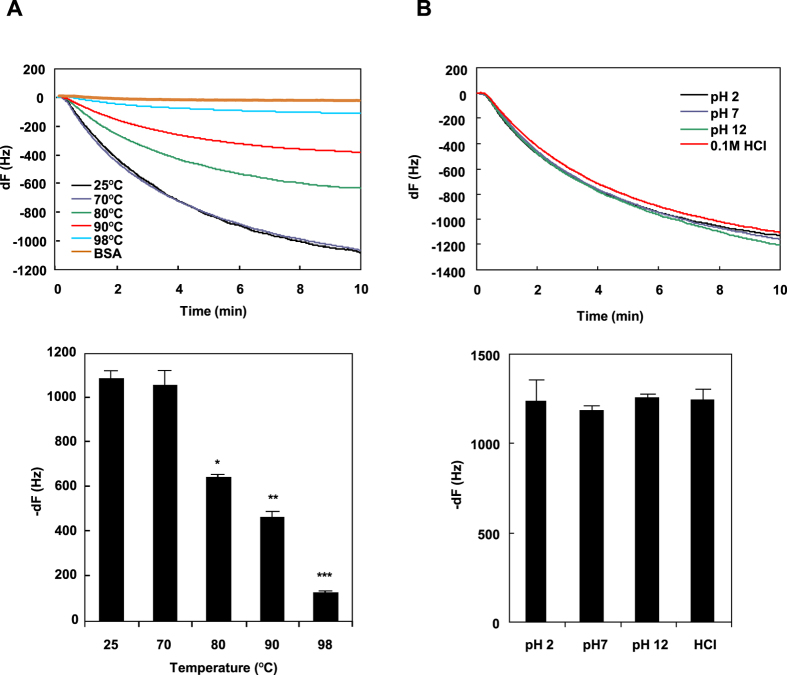
QCM analysis for measurement of the adsorbed mass of AtaA PSD after thermal treatment or acid/alkaline treatment. Upper panels show the frequency changes (dF) observed during the adsorption of AtaA PSD to the gold coated sensor chip after treatments at the indicated temperatures (**A**) and pHs (**B**). Lower panels show frequency changes (dF) at 10 min of the adsorption period shown in (**A**). Data are expressed as the mean ± SEM of three independent measurements. Significant differences from the result obtained at 25 °C, analyzed using Student’s t-test, are indicated by one (p = 0.0089), two (p = 0.0007), or three (p = 0.00006) asterisks.

Obtaining purified AtaA PSD allowed us to directly measure the interaction of the protein molecule with a surface. Quartz crystal microbalance (QCM) is a highly sensitive and label-free mass measurement sensor, which enables quantifying molecules interacting with a quartz crystal sensor chip as a frequency change. We examined the adhesiveness of intact and denatured AtaA PSD using QCM. As shown in Fig. 7A, the resonance frequency rapidly declined with time when AtaA PSD pre-treated at 25 or 70 °C was applied to the QCM sensor. This implies the rapid adhesion of protein molecules to the sensor chip surface. In contrast, the decline in the resonance frequency was faint when bovine serum albumin was applied, emphasizing the stickiness of AtaA PSD. The decline in resonance frequency decreased with increasing treatment temperature for denaturation of the protein molecules and became faint at 98 °C. The bar chart in Fig. 7A shows the frequency change (dF) caused by the adhesion of protein molecules to the QCM sensor when the frequency-shift curve became almost flat (10 min). The absolute value of the dF was almost the same after incubation at 70 °C as it was at 25 °C; it suddenly diminished at 80 °C, gradually diminished with increasing treatment temperature, and became very small at 98 °C, implying that adhesiveness was almost lost in AtaA PSD that was denatured at the high temperature. However, treatment with acidic or alkaline solution did not affect its adhesiveness to the QCM sensor chip at all, and thus the dF remained the same (Fig. 7B). These QCM data suggest that AtaA’s high adhesiveness to an abiotic surface requires its structural integrity. To determine whether the heat denaturation that causes the loss of adhesiveness degrades the AtaA polypeptide itself, AtaA PSD samples after thermal treatment were subjected to sodium dodecyl sulfate polyacrylamide gel electrophoresis (SDS-PAGE). This revealed that the molecular sizes of the monomers in these AtaA PSD samples were unchanged, implying no degradation of the polypeptide by thermal treatment (Fig. S3). Therefore, loss of the adhesive function of AtaA PSD must have been caused by disruption of the 3D structure of the protein.

## Discussion

TAAs have most often been studied using fragmented recombinant proteins expressed in *Escherichia coli*^9^,^42^,^43^,^44^,^45^ or using bacterial cells expressing intrinsic or foreign TAAs on their cell surfaces^6^,^8^,^46^,^47^,^48^. These studies have uncovered the structure and function of TAAs. However, their molecular properties remain unclear because the properties of a fragmented recombinant protein do not always reflect those of the native, full-length molecule. By contrast, proteins isolated using our enzymatic reaping method are guaranteed to be properly folded and assembled because they are actual parts of the native protein secreted by the original carrier cells. As for classical autotransporters that release their PSDs into the extracellular space, PSDs purified from culture supernatants have been used for biochemical characterization^49^ or determination of their crystal structures^50^,^51^. To the best of our knowledge, however, the artificial insertion of a protease recognition site into an autotransporter has never been employed to obtain PSDs for their characterization. In this study, we first inserted a protease recognition site into a TAA to isolate its PSD by proteolytic cleavage, and then we purified the native PSD proteins from the extracellular space. The expected molecular weight from DLS analysis (Fig. 4A) suggested that the AtaA PSD purified by this method appropriately formed a trimer. TEM revealed its nanofibrous structure, which corresponded to the predicted domain configuration having two globular Ylhead domains at the N-terminus of the distal end of the fiber (Nhead) and the C-terminal region near the fiber base (Chead), and a long stalk between the two Ylheads (Fig. 4B). The Chead was not reasonably found on the AtaA PSD short fiber (see Supplementary Fig. S1). These results demonstrate that AtaA PSD was cleaved by HRV 3C protease at the inserted protease recognition site, as designed. The fibrous morphology of the AtaA PSD molecules was disrupted and became difficult to recognize when the AtaA PSD molecules were denatured (Fig. 6A). TEM images of the AtaA PSD obviously changed after incubation of the protein at temperatures over 90 °C, but were unchanged after incubation at temperatures lower than 80 °C. When the temperature exceeded 80 °C, the CD spectrum changed drastically and the ellipticity at 222 nm indicated that the α-helix content decreased sharply, implying drastic denaturation of the protein that resulted in the disruption of its fibrous structure.

In denaturation studies, AtaA PSD showed high structural stability under conditions of extreme pH. It was reported that TAAs maintain their trimeric structure under the standard sample preparation conditions used for SDS-PAGE, and that dissociation into monomers requires boiling in 6–10 M urea solution, 95% formic acid, or extraction with hot phenol^9^,^52^,^53^,^54^. Compared with the other TAAs reported in these articles, AtaA does not seem so resistant to high temperatures. However, it should be noted that the AtaA PSD does not contain a TM, which is important for trimer stability^53^. Nevertheless, AtaA PSD showed thermal stability that is considerably higher than that of typical proteins. Recently, we determined the X-ray crystal structure of the C-terminal PSD of AtaA, which exhibits the toughness of AtaA nanofibers^27^. The crystal structure revealed that interchain interactions of the three polypeptides forming the trimer, especially topological chain exchange, contribute to the toughness, which also should give the AtaA PSD high stability. The high stability is also significant for utilization of AtaA in our newly developed method for microbial immobilization^24^,^25^. The robustness due to the stability is expected to become an advantage in bioprocesses for industrial use.

QCM measurements revealed that AtaA PSD denatured by thermal treatment at high temperatures considerably decreased its adhesiveness (Fig. 7A). By contrast, alkaline and acid treatments did not affect the adhesiveness of AtaA PSD at all (Fig. 7B). However, the heat denaturation that caused the loss of adhesiveness did not degrade the AtaA polypeptide itself (see Supplementary Fig. S3); only the higher-order structure was disrupted. These results lead us to conclude that the 3D structure is essential for the AtaA molecule to exhibit its unique adhesive character. The uniqueness of the adhesive character of AtaA is its nonspecific interactions, which have no specific ligand. In specific interactions, the stereostructure of the binding site for the ligand of a protein is important. In fact, TAAs require stable trimer formation to exhibit specific adhesion to host cells and tissues^52^,^53^. In contrast, nonspecific interactions do not necessarily require such precise stereostructures. Generally speaking, unfolded polypeptides are believed to exhibit nonspecific adhesiveness or a broad binding specificity due to their disordered structures, which allow enlargement of the capture radius and surface exposure of amino acid residues, depending on surface properties. Especially, large polypeptides are thought to be sticky due to multipoint adsorption on an extensive interface^55^,^56^,^57^. It has been speculated that the huge size of AtaA is responsible for its high adhesiveness. This idea was ruled out by the results showing that AtaA PSD almost completely lost its adhesive character after treatment at 98 °C, despite maintaining the full length of the polypeptide. It should be stressed that maintaining a proper 3D fiber structure is crucially important for the high, nonspecific adhesiveness of the AtaA molecule, and that the adhesiveness cannot be achieved from the large size of the protein alone.

In conclusion, we established a methodology that uses specific protease digestion to prepare the very large PSD of AtaA while retaining its native properties. We observed, for the first time, the molecular shape of purified AtaA PSD and found that its fibrous structure has high stability in strict pH conditions. The 3D structure forming this fiber is necessary for AtaA PSD to exhibit its nonspecific adhesiveness.

## Methods

### Bacterial strains and culture conditions

The bacterial strains used in this study are listed in Supplementary Table S1. *Acinetobacter* sp. Tol 5 and its derivative strains were maintained as previously described^22^. Luria-Bertani (LB) medium was supplemented with ampicillin (500 μg/mL) and gentamicin (10 μg/mL), when required. Arabinose (0.5%, wt/vol) was added to induce expression of *3CataA* and *3CFGG2ataA*. Production of the expressed proteins was confirmed by SDS-PAGE, followed by immunoblotting using anti-AtaA_699–1014_ antiserum, as previously described^22^.

### Construction of p3CAtaA and p3CFGG2AtaA

The procedures for the construction of p3CAtaA and p3CFGG2AtaA, which were used to generate AtaA with an inserted HRV 3C protease recognition site, are schematically shown in Supplementary Figs S4 and S5, respectively. Insertion of the sequence encoding the protease recognition site into *ataA* was performed using overlap-extension PCR. The primers used in this study are listed in Supplementary Table S2.

To construct p3CAtaA, an initial PCR was performed with template pTA2::*ataA* vector and two primer sets: Bgl II ataA-F/HRV3C ataA-R and HRV3C ataA-F/Xba I ataA-R. A second round of PCR was performed using the DNA fragments amplified by the first PCR as templates using the primer set Bgl II ataA-F/Xba I ataA-R. The amplicon was inserted into the vector pTA2, generating pTA2::*3CataA*-fragment. After DNA sequencing for confirmation, the *3CataA* fragment was excised again with *Bgl* II and *Xba* I and substituted for the *Bgl* II-*Xba* I fragment of pTA2::*ataA*, generating pTA2::*3CataA*. Thus, the *3CataA* gene was constructed, and then sub-cloned into the pARP3 vector^22^, generating p3CAtaA. Transformation of the Δ*ataA* mutant Tol 5 4140^38^ was carried out by conjugal transfer from *E. coli* S17-1 strain^58^, as previously described^22^.

To construct of p3CFGG2ataA, plasmid pDONR::*ataA* was digested with *Kpn* I and the linearized plasmid was re-circularized by self-ligation. The resulting circular plasmid was digested with *Xcm* I and the linearized plasmid was again re-circularized by self-ligation, generating pDONR::*ataA* (Δ*Xcm* I, *Kpn* I). This plasmid was used as the template for overlap PCR with two primer sets: In-Fusion FGG2-F/3CataA-FGG2-R and 3CataA-FGG2-F/In-Fusion FGG2-R. A second round of PCR was performed using DNA fragments amplified by the first PCR as templates and the primer set In-Fusion FGG2-F/In-Fusion FGG2-R. The amplicon was fused to pDONR::*ataA* (Δ*Xcm* I, *Kpn* I), which was linearized by double digestion with *Xcm* I and *Kpn* I using the In-Fusion HD cloning Kit (Takara Bio, Shiga, Japan), generating pDONR::*3CFGG2ataA* (Δ*Xcm* I, *Kpn* I). Finally, two *ataA* gene fragments, a *Kpn* I fragment and an *Xcm* I fragment, were inserted into the *Kpn* I and *Xcm* I restriction sites of pDONR::*3CFGG2ataA* (Δ*Xcm* I, *Kpn* I), generating pDONR::*3CFGG2ataA*. Thus, *3CFGG2ataA* was constructed and then sub-cloned into the vector pARP3^22^, generating p3CFGG2ataA.

### Obtaining AtaA passenger domain by protease digestion

Bacterial cells were harvested by centrifugation, suspended in 10 mL of 50 mM HEPES buffer (pH 7.0) supplemented with 80 units of HRV 3C protease (Merck Millipore, MA, USA), incubated at 4 °C for 2 days in a Proteosave SS 15-mL centrifuge tube (Sumitomo Bakelite, Tokyo, Japan), and then centrifuged at 10,000 × *g* at 4 °C for 10 min. The 10 mL of supernatant was dispensed into new Proteosave SS 1.5-mL microtubes and fractionated by precipitation in the 30% solution of the saturated concentration of ammonium sulfate at a room temperature. The precipitate was dissolved in dH_2_O and subjected to ultrafiltration with a 100 kDa molecular weight cut-off filter (Amicon Ultra; Merck Millipore) by centrifugation at 10,000 × *g* and 4 °C. The residual AtaA PSD was washed several times with dH_2_O and finally concentrated in a final volume of 50 μL of dH_2_O solution. The purity of this AtaA-PSD sample was confirmed by SDS-PAGE followed by Coomassie Brilliant Blue (CBB) staining.

### Separation of AtaA passenger domain by sucrose density gradient centrifugation

Purified AtaA PSD (100 μL) was layered on a linear sucrose gradient (5 to 50% sucrose in dH_2_O) prepared using a gradient maker (Hitachi Kouki, Tokyo, Japan) in a 13-mL ultracentrifuge tube (PA13; Hitachi Kouki). This tube was centrifuged at 200,000 × *g* for 18 h. Gradient fractions of 1 mL were collected from the top of the tube (11 fractions in total) and 30 μL of sample from each aliquot was subjected to SDS-PAGE and CBB staining.

### Cell analysis

Flow cytometric analysis of cell-surface 3CAtaA was performed as previously described^22^, with slight modifications. Before fixation with 4% paraformaldehyde solution, pre-fixation was conducted by mixing 4% paraformaldehyde and suspension culture in a 1:1 ratio. The cells were washed with dH_2_O after fixation. FlowJo software (Tomy Digital Biology, Tokyo, Japan) was used to create histograms and to compare the fluorescence intensities among the samples. For CLSM, the same cell samples immunoreacted with the anti-AtaA_699–1014_ antiserum as the first antibody and Alexa Fluor 488-conjugated anti-rabbit antibody as the secondary antibody were placed onto a slide glass for 15 min. After being washed with NET buffer (150 mM NaCl, 5 mM EDTA, 50 mM Tris-HCl, 0.05% Triton X-100; pH 7.6) twice and dH_2_O once, cells were observed under a CLSM (FV1000, OLYMPUS, Tokyo, Japan).

Adherence assays were performed as previously described^23^, with slight modifications. Aliquots (200 μL) of a bacterial cell suspension in BS-N medium^23^ at an optical density at 660 nm of about 0.5 were placed into a 96-well PS plate (353072; Becton, Dickinson and Company, NJ, USA) or a 96-well glass plate (FB-96; Nippon Sheet Glass Co., Ltd., Tokyo, Japan). After incubation for 2 h at 28 °C, cells adhering to the well surface were stained with 1% crystal violet solution for 15 min. The stain was eluted from the cells with 70% ethanol solution, and the A_590_ of the eluate was measured using a micro plate reader (ARVO X3; PerkinElmer, MA, USA).

### Biophysical and biochemical analyses of the AtaA passenger domain

The DLS of purified AtaA PSD in dH_2_O (0.09 mg/mL) was measured using a Zetasizer Nano ZSP (Malvern Instruments, UK), yielding the particle size distribution per volume data and their average hydrodynamic diameter. The relationship between hydrodynamic diameters and molecular weights is expressed by the following equation:


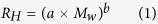


where *R*_*H*_ is a hydrodynamic diameter measured by DLS, *Mw* is a molecular weight and a, b are coefficients fixed by a molecular type: globular protein, linear polymer, or dendrimer. The molecular weight of AtaA PSD distributed in particles of different sizes was estimated as a linear polymer by using this relationship embedded in Zetasizer Nano software (Malvern Instruments).

To conduct electron microscopy on purified AtaA PSD nanofibers, 10 μL of the sample solution (3–30 μg/mL) and 2 μl of 50 mM KCl solution were placed onto a copper grid with an ultra-high resolution carbon substrate (UHR-C10; Okenshoji, Tokyo, Japan). The sample was hydrophilized by plasma treatment using a plasma ion bombarder (PIB-10; Vacuum Device, Ibaraki, Japan) immediately before use. When observing AtaA PSD treated at different pHs, 1 μL of 1 M Tris-HCl (pH 6.8) was added to each 10 μL sample before dropping on the grid. The grids were then incubated for 10 min at room temperature to allow adsorption of the proteins and washed by dipping 20 times into 10 mM KCl solution and three times into dH_2_O. Negative staining of the proteins on the grid was performed with the organo-tungstate compound Nano-W (Nanoprobes, NY, USA). These sample preparation procedures were conducted in a moisture box to avoid desiccation. The specimen was observed using a TEM system (JEM-1400EX; JEOL, Tokyo, Japan) operated at 100 kV.

For denaturation analysis of AtaA PSD at different pHs, buffers of pH 2, 7, and 12 were prepared by mixing 50 mM citric acid solution, 200 mM boric acid solution, and 100 mM sodium phosphate. AtaA PSD in dH_2_O and the above-mentioned buffers were mixed in a 19:1 ratio (final concentration of AtaA was 0.15 mg/mL) on micro-sampling discs for CD spectroscopy. For the extreme low pH condition, 0.1 M HCl solution was used instead of the buffers. For denaturation by thermal treatment, 0.15 mg/mL of AtaA PSD in dH_2_O was incubated in a Proteosave SS 1.5-mL microtube at different temperatures for 5 min. The heat-treated samples were then centrifuged for 1 min at 15,000 × *g* and 4 °C to remove debris, and the temperature of the samples was brought back to a room temperature before conducting further studies.

The CD spectrum of purified AtaA PSD was recorded using a J-725 spectropolarimeter (JASCO, Tokyo, Japan). The parameters (wavelength range, scan rate, scan interval, response, and band width) were set at 190–250 nm, 100 nm/min, 0.1 nm, 0.25 s, and 1.0 nm, respectively. For heat-treated samples, 10 μL of the sample (0.15 mg/mL) was applied to the micro-sampling disc after cooling to room temperature. The spectra from the buffer or dH_2_O were recorded as blanks. The α-helix and β-strand contents of AtaA were calculated using the DichroWeb^59^,^60^ program with the CONTINLL algorithm and Protein Reference Data Sets 7.

The adhesiveness of AtaA PSD was measured using a QCM system (AFFINIX Q8; ULVAC, Kanagawa, Japan). A gold coated electrode (QCM01S-01; ULVAC) was cleaned with piranha solution (H_2_SO_4_:30% H_2_O_2_ = 3:1) and equilibrated with 99 μL of phosphate-buffered saline (PBS) (314-90185; Nippon Gene, Tokyo, Japan) at 25 °C. After equilibration, 1 μL of AtaA PSD solution (0.1 mg/mL) was added to 99 μL of PBS on the electrode and the frequency change was measured at 25 °C. Heat-treated AtaA PSD samples and AtaA PSD samples in buffers at various pHs or in 0.1 M HCl were used directly for the QCM measurement.

## Additional Information

**How to cite this article**: Yoshimoto, S. *et al*. An *Acinetobacter* trimeric autotransporter adhesin reaped from cells exhibits its nonspecific stickiness via a highly stable 3D structure. *Sci. Rep.* **6**, 28020; doi: 10.1038/srep28020 (2016).

## Supplementary Material

Supplementary Information
