# Supplementary material for: An Acinetobacter trimeric autotransporter adhesin reaped from cells exhibits its nonspecific stickiness via a highly stable 3D structure
Source: Sci Rep. 2016 Jun 16;6:28020. doi: 10.1038/srep28020 (PMC4910087; doi:10.1038/srep28020)
Supplement: Supplementary Information [file srep28020-s1.pdf]

Supplementary Information

An *Acinetobacter* trimeric autotransporter adhesin reaped from cells exhibits its nonspecific stickiness via a highly stable 3D structure

Shogo Yoshimoto†, Hajime Nakatani†, Keita Iwasaki, and Katsutoshi Hori

Department of Biotechnology, Graduate School of Engineering, Nagoya University, Furo-cho, Chikusa-ku, Nagoya, Aichi 464-8603, Japan

†These authors contributed equally to this work.

Table S1 Bacterial strains and plasmids used in this study

Strain or plasmid	Description	Reference
Acinetobacter sp.		
Tol 5	Wild type strain	25
Tol 5 4140	Unmarked Δ*ataA* mutant of Tol 5	29
Escherichia coli		
DH5α	Host for routine cloning	TaKaRa
S17-1	Donor strain for conjugation	45
Plasmid		
pTA2	Cloning vector, Ap^r	TOYOBO
pARP3	*E. coli-Acinetobacter* shuttle expression vector, araC-P_{BAD}, Gm^r, Ap^r	27
pAtaA	*ataA*-expression vector, pARP3::*ataA*	27
pDONR::*ataA*	*ataA* inserted pDONR	27
pTA2::*ataA*	*ataA* inserted pTA2	This study
p3CAtaA	*3CataA*-expression vector, pARP3::*3CataA*	This study
p3CFGG2AtaA	*3CFGG2ataA*-expression vector, pARP3::*3CFGG2ataA*	This study

Table S2 Primers used in this study

Primer	Sequence (5'→3')†
Bgl II ataA-F	GGTTTGAGCAATAAAGATCTAAATTCAAC
HRV3C ataA-R	GGGTCCCTGAAAGAGGACTTCAAGCCCACCACCAAGATAATTGACTAC
Xba I ataA-R	TGGGTCTAGAGAATTAGTCAATCAC
HRV3C ataA-F	CTTGAAGTCCTCTTTCAGGGACCCGGTGGTGGGGCAGGTTATGACAAC
In-FusionFGG2-F	ACAGCTGACCAAGTTAGTAGTGGG
In-FusionFGG2-R	GAGCCATCTGCATTGGTACC
3CataA-FGG2-F	CCTCTTTCAGGGACCTGGAGGTGGATCAACCACAATTGATGCAACG
3CataA-FGG2-R	CTCCAGGTCCCTGAAAGAGGACTTCAAGTCCACCACCAATTGCATTCTTCACG

†Underlined and double-underlined descriptions represent the DNA sequences of the HRV 3C protease recognition site and the glycine linker, respectively.

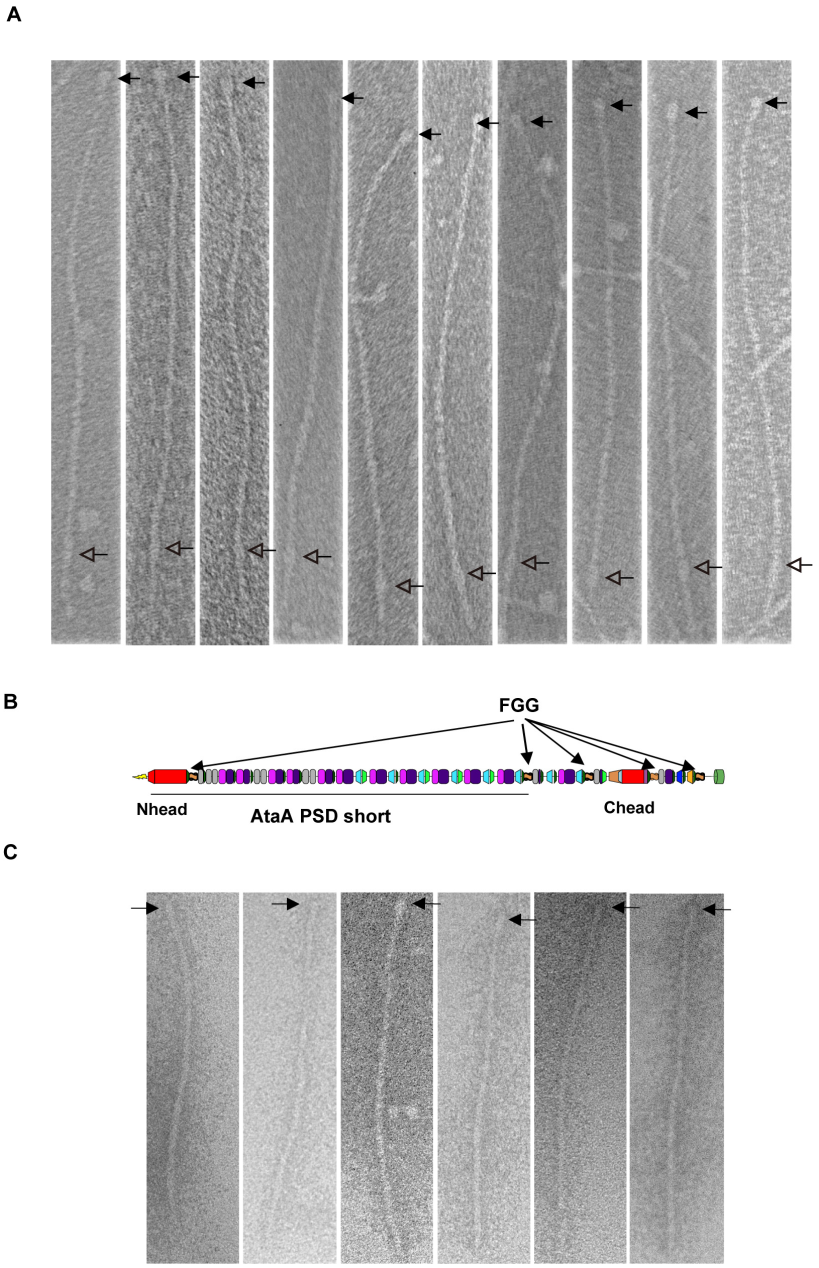

Figure S1. Morphological analysis of AtaA PSD and its short version by TEM. (A) TEM images of negatively-stained isolated AtaA PSD nanofibers. Globular structures at the tip and near the base of the AtaA PSD nanofibers are designated by black arrows and white arrows, respectively. (B) A schematic of the primary structure of AtaA PSD short, which was isolated by proteolytic cleavage at FGG_2. (C) TEM images of isolated AtaA PSD short nanofibers. Globular structures can be observed only at the fiber tips (arrows).

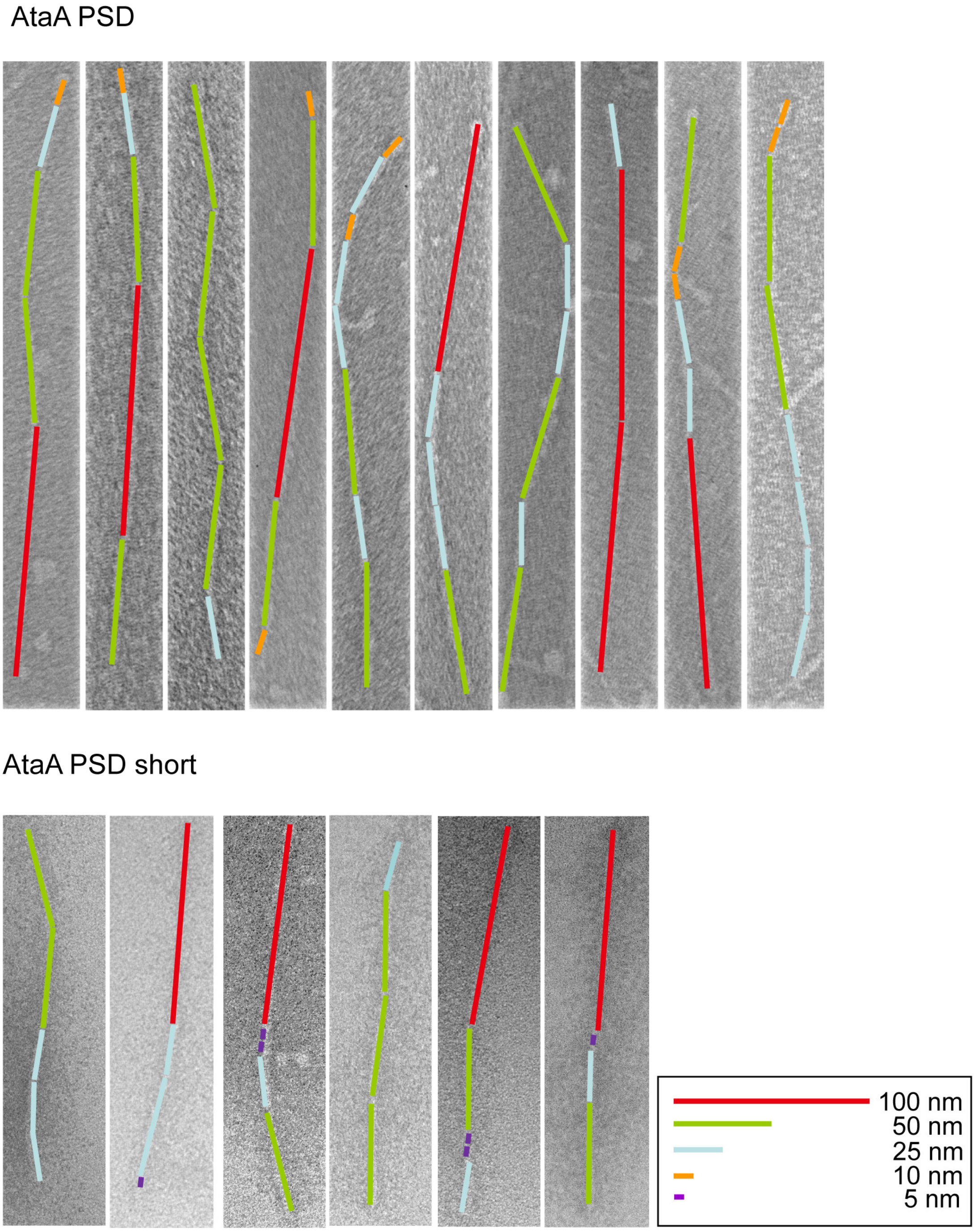

Figure S2. Measurement of the fiber length of purified AtaA PSD and its short version. AtaA PSD nanofibers and AtaA PSD short nanofibers are fitted by scale bars on the images; red, green, blue, orange, and purple bars indicate distances of 100, 50, 25, 10, and 5 nm, respectively.

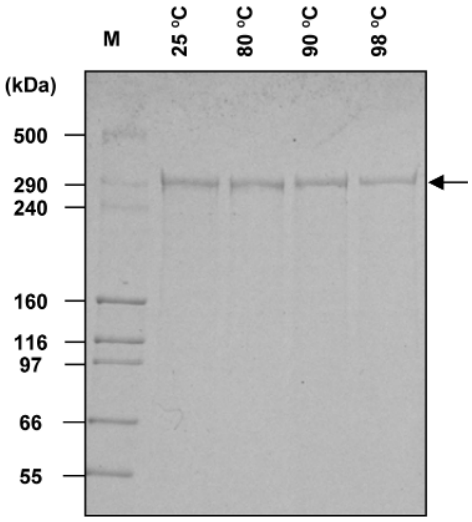

Figure S3. Examination of AtaA PSD degradation after thermal treatment. After thermal treatment, the AtaA PSD samples were separated by SDS-PAGE and the monomeric AtaA PSD was detected by CBB staining. The arrow indicates the full-length monomeric AtaA PSD polypeptides.

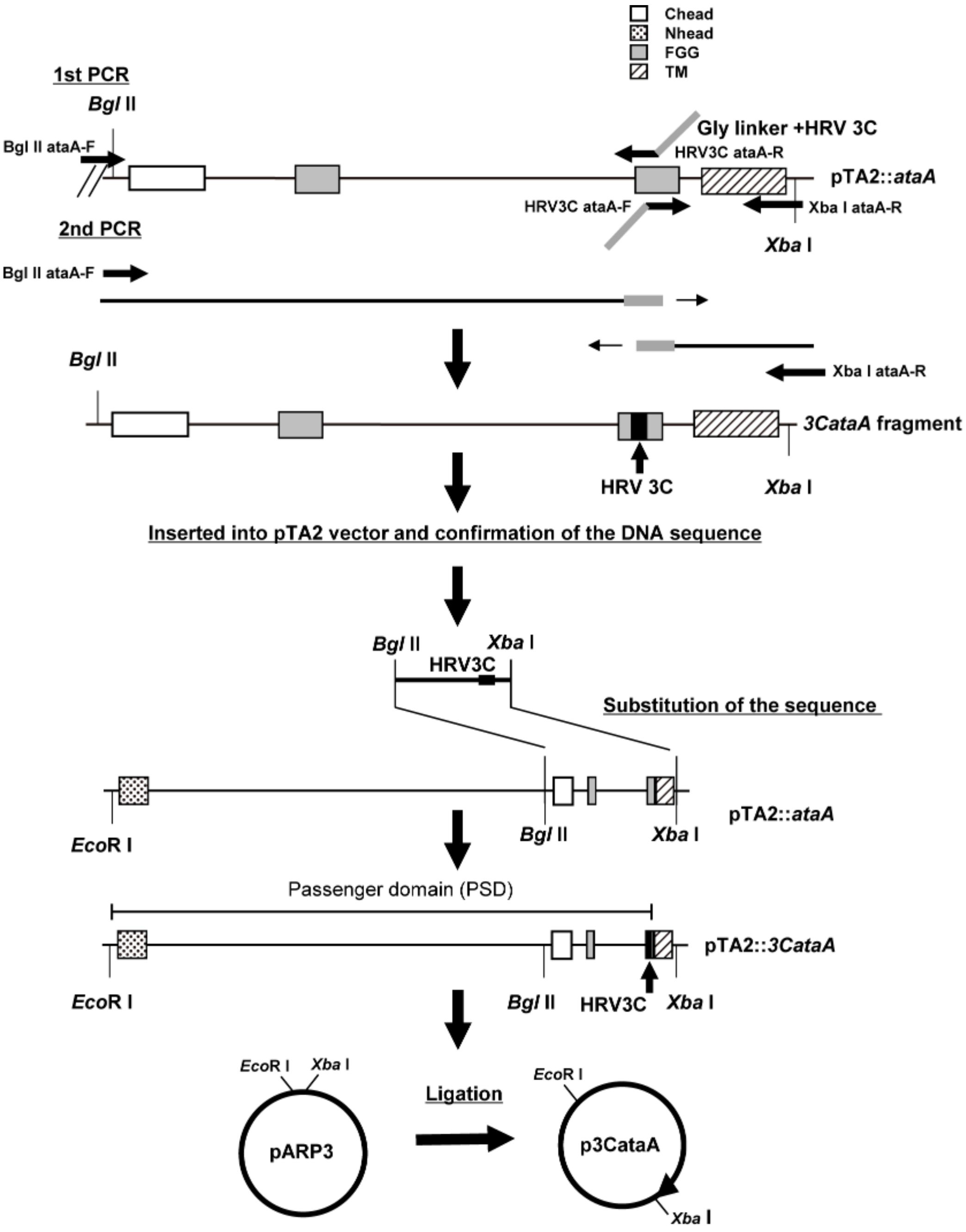

Figure S4. Schematic procedure for the construction of p3CAtaA, which encodes AtaA with an HRV 3C protease recognition site inserted at FGG_5. AtaA PSD has two FGG motifs between the Chead and TM: FGG_4 and FGG_5. FGG_5, which appears nearer to the TM, was the target for insertion of the HRV 3C protease recognition site.

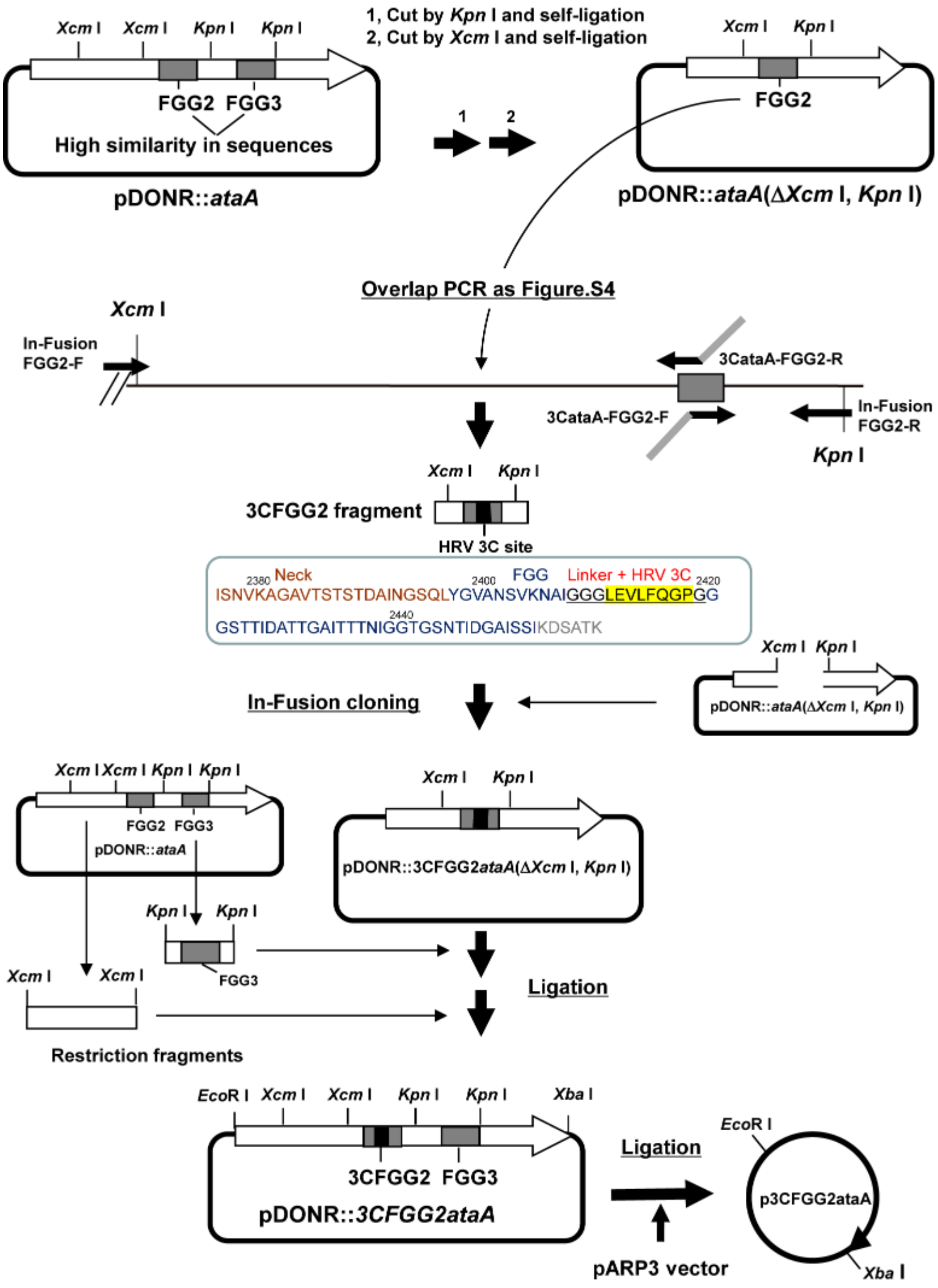

Figure S5. Schematic procedure for the construction of p3CFGG2AtaA, which encodes AtaA with an HRV 3C protease recognition site inserted at FGG_2. AtaA PSD has three FGG motifs between Nhead and Chead: FGG_1, FGG_2, and FGG_3. FGG_2 was the target for the insertion of the HRV 3C protease recognition site.
